# The variety of primary healthcare organisations in Australia: a taxonomy

**DOI:** 10.1186/1472-6963-13-130

**Published:** 2013-04-08

**Authors:** John Rodwell, Andre Gulyas

**Affiliations:** 1 Faculty of Business, Australian Catholic University, Locked Bag 4115 Fitzroy MDC, Victoria, 3065, Australia

**Keywords:** General practice, Health care organisations, Taxonomy, Configurations

## Abstract

**Background:**

Healthcare policy appears to treat healthcare organisations as being homogenous, despite evidence that they vary considerably. This study develops a taxonomy of primary health care practices using characteristics associated with the job satisfaction of general medical practitioners (GPs) and the practices.

**Methods:**

The study used data from 3,662 survey respondents who were GPs in the 2009 wave of the MABEL survey. Cluster analyses were used to determine natural groups of medical practices based on multidimensional characteristics.

**Results:**

Seven configurations of primary health care practices emerged from multivariate cluster analyses: optimised team, independent craft, reactive, winding down, classic, practitioner flexible, and scale efficiency.

**Conclusions:**

This taxonomy of configurations moves beyond simplistic categorisations such as geographic location and highlights the complexity of primary health care organisations in Australia. Health policy, workforce and procedure interventions informed by taxonomies can engage the diversity of primary health care practices.

## Background

Policy makers often treat primary healthcare as consisting of homogenous organisations [1] and are rarely sensitive to local contexts or the variety of practices and contexts [2]. For example, recent health reforms in aimed at better connecting the network of health providers in Australia do not acknowledge the varied forms of general practices that may exist [3]. The main disadvantage of this lack of sensitivity means that policy prescriptions are often too rigid to be effective for all practices. Organisational categorisation systems are a mechanism for policy and management practices to be more sensitive to the variety of organisations across the sector, and initial categorisations proposed three forms: solo, small group, and large group practices [4]. More recent research has demonstrated that there may be as many as six forms of practices; based on size, whether they contain hospital work and whether these practices are multi-disciplinary or conduct community activities [5], although this taxonomy was based on practices in Canada in the 1980s. These attempts at categorising healthcare organisations provide a precedent for grouping practices.

A greater awareness of the diversity in the sector may inform policy decisions enabling those decisions to be more sensitive to this diversity. Therefore, this paper challenges the assumption that primary healthcare organisations are a relatively homogeneous group and argues that they are much more diverse than is usually assumed. Ultimately, this study aims to create greater awareness of the complex nature of the primary health care sector by investigating the nature of a taxonomy based on the aforementioned characteristics of practices, and to determine the viability of a multi-dimensional approach to categorise primary health care practices.

A useful taxonomy of organisations would group organisations with similar characteristics in the same class on multiple dimensions [6] and accurately reflect the reality from which they are derived. Taxonomies with these qualities can be achieved through the development of what is termed “configurations” that holistically reflect how several forms of organisations may emerge from the interaction of parts of an organisation [7]. These configurations can be defined as commonly occurring clusters of characteristics (that is, natural groups of entities that share several similar characteristics that distinguish each group) [8], caused by a stable and complex form of interdependency among characteristics [9].

Characteristics that reflect the multi-dimensional nature of primary health care practices include characteristics of the practice that can be augmented by the predictors of job satisfaction, especially as job satisfaction is integral to decisions to retire from the medical workforce [10] and consequently central to addressing important issues in the sector, such as the medical workforce shortage. Characteristics that affect the satisfaction of GPs include: being part of a multi-physician practice, access to high quality ancillary services, perceived patient complexity and autonomy [11], relationships with colleagues, work variety and not working long hours [12,13]. However, some of these characteristics may vary depending on the context. For example, the complexity of services GPs deliver increases with remoteness and remote practitioners have less support and have more responsibility for their patients’ health [14]. Similarly, female GPs tend to be younger, are more likely to be part-time than male GPs and are less likely to be in remote practices [15]. Multidisciplinary care referrals depend on the GP’s links with allied health professionals and the number of GPs, particularly full-time GPs, in the practice [16]. That is, primary health care practices vary along multiple dimensions, which can be considered in deriving configurations to represent this organisational variety. Together, the commonly-cited characteristics of geography (e.g., rural, metropolitan) and size, in combination with the predictors of job satisfaction, present a wide range of dimensions of primary health care practices. Therefore, this study aims to develop a taxonomy of primary health care practices based on a variety of characteristics to demonstrate the multi-dimensional complexity of primary health care practices. It was hypothesised that several forms of general practices exist and that these forms will cluster into distinct groups based on multi-dimensional similarities within groups and multi-dimensional differences between groups.

## Methods

### Ethics

The Medicine in Australia: Balancing Employment and Life (MABEL) study was approved by the University of Melbourne Faculty of Economics and Commerce Human Ethics Advisory Group (Ref. 0709559) and the Monash University Standing Committee on Ethics Involving Research on Humans (Ref. CF07/1102-2007000291) [17].

### Participants

Data was based on 3,662 GPs from the second wave of MABEL, which is a longitudinal study of Australian doctors, representing a response rate of 64.9% [17]. The GP respondents were 51.8% male, mostly between the ages of 40–59, and most were working in a major city (62.4%), followed by inner regional (22.2%) and outer regional (15.4%) areas.

### Materials

#### Demographics

Respondents indicated the year of their birth, which was used to impute respondents’ age. Gender was imputed using AMPCo data. Respondents also indicated the year they began working at their current practice.

#### Job satisfaction

Job satisfaction was measured using a 10-item scale from the Work and Life Attitudes Survey [18]. Respondents were asked to rate their satisfaction of several features of their current job on a five point scale (from *very dissatisfied* to *very satisfied*). A ‘not applicable’ option was also added. Items included “taking everything into consideration, how do you feel about your job?” and “freedom to choose your own method of working”. This scale has demonstrated good psychometric properties, including good reliability, and has been used in studies of Australian doctors [19]. In the current study, job satisfaction demonstrated a good reliability coefficient (Cronbach’s alpha = .88). Respondents were also asked to rate their self-perceived general health on a five point scale (from *excellent* to *poor*).

The survey used a modified version of the job content questionnaire [18]. The scale asked respondents to indicate the extent they agreed or disagreed with several statements relating to their job, on a five point scale (from *strongly* agree to *strongly disagree*). Individual items in this scale were used in this study to examine respondents’ satisfaction with specific aspects of their current job and work environment. Specifically, items that appeared to relate to work commitments included “the balance between my personal and professional commitments is about right” and “the hours I work are unpredictable”. Items relating to stressor from patients included “my patients have unrealistic expectations about how I can help them” and “the majority of my patients have complex health and social problems”.

#### Place of work

Respondents were asked how many male and female full time and part time GPs (including themselves) currently worked at their main practice, and how many other health workers or professionals were employed in their current main practice (including nurses, administration, allied health, and other staff). Respondents were also asked whether they currently worked in a hospital, whether their current main practice was co-located with other health or welfare professionals and to indicate the percentage of patients that they *bulk bill*, a payment option under the Australian health care system whereby patients are not charged a fee, but rather the government pays a proportion of the fee directly to the health care provider.

One item asked whether respondents worked after hours or on-call themselves.

#### Geographic location

Respondents were asked the location of their main place of work. These responses were grouped into five categories indicating degree of remoteness: major city, inner regional, outer regional, remote and very remote. Due to the low amount of responses of GPs classified as remote or very remote, these GPs were re-coded into the outer regional category [17].

To avoid identification of individuals that provided more easily identifiable responses, several variables were top-coded to equal the threshold for the highest values in the data so that these responses could be used: full time male GPs and female part time GPs (12+), part time male GPs (8+), full time female GPs (7+), nurses (12+), allied health employees (12+), administration employees (22+), and other (10+) employees [17].

### Data analysis

Cluster analyses were used to determine patterns of characteristics occurring among the GP’s perceptions of their practices. A weighted variable was created to amalgamate number of full time GPs and part time GPs. The weighted variable was made by adding number of part time GPs, multiplied by 0.6, to the number of full time GPs. Analyses were conducted to find the ratios of mean hours of respondents working less than 40 hours per week (i.e. part time GPs), to the mean hours of all respondents and to the mean of respondents working 40+ hours per week (i.e. full time GPs). These ratios ranged from approximately 0.5 to 0.7, and the average of these ratios (0.6) was used as the multiplier to weight part time GPs. The ratios were checked using sensitivity analyses that varied the 0.6 weight from 0.5 up to 0.7, and changing the weighting within this range did not substantively change the results. To avoid excessive weighting of clusters towards the size of the practice, ratio variables were created by dividing the number of nurses by the weighted GP variable, and the number of administration staff by weighted GPs.

The cluster analyses used SPSS in a two-stage approach (as recommended by Hair et al. [20]) that increases the validity of solutions. First, hierarchical cluster analyses using Wards’ clustering methods and squared Euclidean distances suggested three-, four-, five-, six-, seven- and eight-cluster solutions. The means from the hierarchical solutions were then used as the starting means for the second stage, k-means non-hierarchical clustering.

Cluster analyses require further interpretation once they have been established. Post-hoc analyses were conducted by one-way analyses of variance (ANOVAs) and chi-squared tests on the seven-cluster solution to enable further interpretation of the cluster solutions, but not specifically for inferential purposes, as recommended by [21]. However, although most of the variables had unequal variances between clusters, conducting non-parametric tests produced the same results and subsequently only the parametric results are reported. To ensure that only the discriminating variables that defined the respective clusters were noted, G*Power3 [22] was used to derived alpha (.001). All chi-squared, ANOVA and LSD post-hoc tests were significant and the means or proportions for the variables are in Table 1.

**Table 1:** Means, percentages and post-hoc results for the clusters

	**1**	**2**	**3**	**4**	**5**	**6**	**7**	**Post-hoc**^**a**^
**Practice characteristics used in cluster analyses**								
The IT systems I use are very helpful in day-to-day practice†	2.87	3.26	3.15	1.18	3.22	3.09	2.98	2,3,5 > 1,7 > 4,2,5 > 6 > 4
My patients have unrealistic expectations about how I can help them†	2.19	1.38	2.61	2.34	3.19	1.42	2.21	5 > 3 > 1,4,7 > 2,6
The majority of my patients have complex health and social problems†	3.01	3.27	3.29	3.09	3.16	1.41	2.64	2,3 > 1,4 > 7 > 6,5 > 6,7
The hours I work are unpredictable†	2.03	0.95	3.26	1.59	1.13	0.98	1.29	3 > 1 > 4 > 5,7 > 2,6
Weighted GPs †	1.51	4.70	4.38	4.55	4.51	4.72	11.95	7 > 2,3,4,5,6 > 1
Nurse to GP ratio†	2.44	0.48	0.53	0.49	0.49	0.46	0.37	1 > 2,3,4,5,6 > 7
Administration staff to GP ratio†	3.34	1.10	1.21	1.06	1.15	1.14	0.78	1 > 2,3,4,5,6 > 7
Percentage of patients bulk billed†	75.55	56.55	62.61	71.85	61.30	49.69	66.55	1,4 > 2,3,5 > 6,7 > 2 > 6
Collocated†	39.1%	43.7%	40.2%	43.5%	40.1%	44.2%	68.2%	
On-call†	70.9%	45.4%	71.5%	47.5%	47.7%	40.9%	63.4%	
**Other practice characteristics**								
Remoteness								
*City*	33.3%	62.6%	46.1%	60.5%	65.8%	68.0%	72.9%	
*Inner regional*	22.2%	25.6%	31.7%	23.1%	22.2%	18.6%	18.5%	
*Outer regional*	44.4%	11.8%	22.2%	16.4%	12.0%	13.4%	8.6%	
Hospital	35.5%	21.0%	47.5%	22.8%	21.1%	20.1%	22.9%	
Full Time GPs (zeroed)	0.92	2.81	2.97	2.69	2.72	2.79	8.84	7 > 2,3,4,5,6 > 1
Part Time GPs (zeroed)	0.99	3.14	2.34	3.09	3.00	3.22	5.17	7 > 2,4,5,6 > 3 > 1
Number of allied health professionals	2.47	1.40	1.23	1.68	1.21	1.18	2.40	7 > 2,3,4,5,6,1 > 2,3,5,6
Number of administration	4.63	4.87	4.90	4.48	4.80	4.98	9.27	7 > 1,2,3,4,5,6
Number of nurses	3.71	2.24	2.24	2.23	2.18	2.12	4.35	1,7 > 2,3,4,5,6
Number of other staff	3.32	1.55	1.44	2.67	1.67	1.05	3.38	1,4,7 > 2,3,5,6
Male GPs	64.5%	44.9%	63.0%	53.5%	47.3%	48.4%	59.9%	
Age								
*44 or under*	38.6%	33.3%	24.7%	31.9%	37.3%	44.5%	34.8%	
*45-59*	39.4%	51.5%	59.6%	47.4%	49.6%	42.3%	50.8%	
*60+*	22%	15.1%	15.7%	20.8%	13.2%	13.5%	14.4%	
**Job Aspects**								
The balance between my personal and professional commitments is about right	1.94	2.56	1.63	2.09	2.06	2.68	2.31	2,6 > 4,5,7 > 3,2,6 > 1
Running my practice is stressful most of the time	2.26	1.87	2.76	2.32	2.42	1.52	1.93	3 > 4,5 > 2,7 > 6,3 > 1 > 2 > 6
Total hours worked per week	47.98	35.99	48.61	38.44	39.06	35.29	38.62	1,3 > 2,4,5,6,7
**Outcomes**								
Job satisfaction	30.41	32.76	28.94	28.89	30.27	32.55	31.03	2,6,7 > 3,4,5; 2 > 7
Self-rated general health (reverse-scored; low = healthier) ^b^	1.13	0.89	1.20	1.17	1.13	0.86	1.03	3,4,5 > 2,6
Tenure (years, excl. years tenure = 0)	8.99	10.18	12.33	9.98	10.08	8.16	9.26	3 > 2,4,5,6,7
**Number of GPs in cluster**	110	535	478	324	461	548	314	

† Denotes variables used in cluster analyses. ^a^Post-hoc refers to the significant differences between clusters. ^b^For self-rated general health 0 = excellent, 4 = poor.

Note. The clusters were the same whether listwise deletion or missing data imputation was used. The more conservative listwise approach was used.

Cases were excluded if the value for the weighted variable was zero, as these respondents erroneously specified that no GPs, including themselves, were working at their current place of work (n = 395). The main argument against listwise deletion to handle missing data is that it may bias results e.g. [23]. To demonstrate that there was minimal bias from using listwise deletion to handle the missing data several methods to address missing data were used and the results of the analyses based on each of the methods were compared. Three sets of analyses were conducted. First, listwise deletion was applied to all variables used in the cluster analysis. Second, missing data imputation, using expectation maximisation with a normal distribution was applied to the continuous variables. Third, expectation maximisation was used to impute missing data imputation for continuous and categorical variables. The characteristics of the clusters provided by the results from each of these datasets were the same whether listwise deletion (n = 2770), imputation of continuous variables (n = 3190), or imputation of continuous and categorical variables (n = 3267) was used, demonstrating that listwise deletion did not bias the results. Missing data was excluded on a listwise basis because it is a more conservative approach to dealing with missing data e.g. [24] and more clearly delineates the boundaries of generalizability of the results.

To check for systematic differences, excluded cases missing one or two items (n = 627) were compared with cases included in the analyses. Although the two groups were similar, the ‘missing’ group had smaller practices, as indicated by the weighted number of GPs (M = 3.09, SD = 3.38), than included cases (M = 5.29, SD = 3.41; t(3395) = 14.63, p < .001). The ‘missing’ group also found IT less useful in day-to-day work (M = 2.76, SD = 0.97) than included cases (M = 2.91, SD = 0.93; t(807) = 3.44, p < .001. Further analyses revealed that 25.7% of the ‘missing’ group reported that they currently worked in solo/very small practices (i.e. either one or two full time GPs, one, two or three part time GPs, or one full time and one part time GP). Consequently, 31.5% of solo GPs across the entire dataset had been excluded. However, very small practices were still well-represented in the dataset, as the majority of these were included in the cluster analyses (68.5%), and the items with missing values were fundamental to the analyses.

For the cluster analyses, z-scores were created from the following variables; “the IT systems I use are very helpful in day-to-day practice”, “my patients have unrealistic expectations about how I can help them”, “the majority of my patients have complex health and social problems”, “the hours I work are unpredictable”, weighted number of GPs, ratio of nurses to GPs, ratio of administration employees to GPs, and “approximately what percentage of patients do you bulk bill”. The following variables were left as unstandardised dichotomous variables; “do you do an after-hours or on-call yourself” and “is your current main practice co-located with other health or welfare”.

## Results

No outliers or collinearity were present for the variables used in the cluster analyses, and the treatment of data using listwise deletion did not bias the results. The seven cluster solution, as illustrated in Table 1, was deemed best for several reasons. For example, the agglomeration coefficients identified several possible cluster solutions (i.e. 4, 5, 6, 7 and 8 clusters), which were each subsequently explored with non-hierarchical cluster analyses, following Hair et al. [23]. The seven cluster solution allowed enough detailed separation between the clusters for adequate distinctiveness and discrimination between them, while also being parsimonious without being overly complex. Additionally, these seven clusters seemed to make the most sense and were more easily interpretable compared to the other solutions regarding the types of general practices that may exist.

Based on the means of several practice characteristics for each cluster, the clusters were labelled:

1. **Optimised Team**, encompassing 3.8% of cases, represents small, remote practices, with high nurse to GP, and administration staff to GP, ratios, a high amount of bulk billing, a high amount of on call and GPs at these practices tend to work more hours. These practices also tend to be attached to hospitals.

2. **Independent Craft**, containing 21.4% of cases, represents practices that find IT more useful, with more predictable hours, patients that are on the lower end of having unrealistic expectations, but also patients that have more complex issues and GPs that find running their practice on the lower end of stressful, and have less on call.

3. **Reactive**, comprising 17.4% of cases, represents inner regional practices, that tend to be attached to hospitals, with patients that are complex with unrealistic expectations, and GPs that find running their practice stressful, find IT the most useful for day-to-day activities, have unpredictable hours, the most on call and the highest tenure.

4. **Winding Down**, representing 11.6% of cases, is mostly defined by GPs finding IT least useful in day-to-day practice, and are on the upper end of bulk billing and tend to be older.

5. **Classic**, classifying 16.1% of cases, is defined by having patients with unrealistic expectations and complex issues and GPs that find IT more useful.

6. **Practitioner Flexible,** comprising 17.9% of cases, have better patients, more predictable hours, and the least bulk billing and on call. These practices also tend to be less remote and not hospital linked.

7. **Scale Efficiency**, containing 11.8% of cases, have the most GPs, the smallest admin-to-GP and nurse-to-GP ratios, are on the upper end of bulk billing, and on the upper end of on-call. Also, they have a high percentage of co-location, are much less remote and are the least likely to be attached to a hospital.

Independent Craft practices also had the lowest proportion of male GPs (44.9%) than other groups. Independent Craft practices and Practitioner-flexible practices had better work life balance and self-rated general health than other clusters, whereas Reactive practices had the lowest work life balance. Reactive practices also found running their practices the most stressful relative to the other groups, whereas Practitioner-flexible practices were the least stressed. Further, Independent Craft, Practitioner-flexible and Scale Efficiency practices were the most satisfied with their jobs.

## Discussion

This study successfully derived a taxonomy of primary health practices based on configurations of the characteristics of practices and extended prior research that had noted certain characteristics being important to GP satisfaction e.g., per [11-13]. The seven multi-dimensional configurations derived from the current study move beyond simplistic characterisations such as geography and are similar to the six types of practices outlined by Williams et al. [5], and demonstrate the viability of a multidimensional approach to primary health care practices and the complexity of the sector. The results supported the hypothesis that there are several forms of primary health care practices, and these practices are similar within groups and differentiated from each between groups other along multiple dimensions.

These configurations also suggest how some of the contradictory results in the literature may have emerged. For example, research has reported high satisfaction levels of GPs in rural practices [25], yet there also appears to be a substantial shortage of rural GPs in Australia [26]. In the current study, GPs in clusters 2 (Independent Craft), 6 (Practitioner-flexible) and 7 (Scale Efficiency) had higher levels of job satisfaction than the other clusters. Yet all of the clusters included some degree of rural and regional GPs. The likely cause of differences between types of practices, such as job satisfaction, is multi-dimensional even when comparing only a few clusters, thereby requiring a policy approach that moves beyond simplistic classifications such as a reliance on metropolitan-rural distinctions. The multidimensional approach demonstrated by this study suggests that, rather than examining predictors of job satisfaction across the whole sector, research should account for the various contexts that may influence GP satisfaction.

The main limitation of this study was that approximately one sixth of cases were excluded due to missing or erroneous data. Further analyses revealed that around 25.7 percent of excluded respondents were in solo/very small practices and consequently a small, but notable proportion of these GPs were excluded. To avoid this issue in future, research will need to make greater efforts to sample solo GPs. However, very small/solo practices were still well represented in the results, with 68.5 percent of the very small practices in the cluster analyses.

The taxonomic approach used in the current study can be used in future research to further examine the different needs and contexts of each configuration to gain a greater understanding that would enable policy to be more sensitive to these multidimensional differences across the sector. For example, the specific contexts of each configuration may affect the quality of care they can provide, rather than focussing on simple distinctions such as differences between rural and city, or large versus small, practices. Avenues other than policy prescriptions could also be examined, such as the effectiveness of different management styles or different staffing or funding needs depending on the specific nature of each configuration. Although this taxonomy was developed in Australia, this study has demonstrated the process of how a configurational approach can be applied to health care practices and researchers and policy analysts internationally could examine the configurations of practices to inform policy and other interventions in their respective countries.

## Conclusions

Applying a multidimensional taxonomy when designing health policies could prevent primary healthcare being treated as a homogeneous group, particularly by focusing on issues such as service organisation and role delineation [27], which should potentially enable the development of policies and related interventions (i.e. education, training, allocation of resources), to cater for the variety of organisations as they occur in the sector. The resulting policies may be smaller in scope and would require customisation to the nature of the target cluster(s), but may be more compatible with the pattern of characteristics of the cluster.

## Competing interests

The authors declare that they have no competing interests.

## Author’s contributions

JR conceptualised the study. AG and JR analysed the data. JR led on writing the paper. Both authors read and approved the final manuscript.

## Pre-publication history

The pre-publication history for this paper can be accessed here:

http://www.biomedcentral.com/1472-6963/13/130/prepub
